# 3 Cases of Superior Vena Cava Syndrome Following Percutaneous Right Ventricular Assist Device Placement

**DOI:** 10.1016/j.jaccas.2021.09.005

**Published:** 2021-11-03

**Authors:** John R. Vaile, J. Eduardo Rame, Rene J. Alvarez, Howard T. Massey, Vakhtang Tchantchaleishvili, Alec Vishnevsky, Indranee N. Rajapreyar, Yevgeniy Brailovsky, Mahek K. Shah

**Affiliations:** a Sidney Kimmel Medical College, Thomas Jefferson University, Philadelphia, Pennsylvania, USA; b Department of Heart Failure and Transplant, Thomas Jefferson University Hospital, Philadelphia, Pennsylvania, USA; c Department of Cardiothoracic Surgery, Thomas Jefferson University Hospital, Philadelphia, Pennsylvania, USA; d Department of Interventional and Structural Cardiology, Thomas Jefferson University Hospital, Philadelphia, Pennsylvania, USA

**Keywords:** cardiac assist devices, cardiac transplant, imaging, percutaneous coronary intervention, CVP, central venous pressure, ICD, implantable cardioverter defibrillator, LVAD, left ventricular assist device, OHT, orthotopic heart transplantation, RVAD, right ventricular assist device, SVC, superior vena cava, TEE, transesophageal echocardiogram

## Abstract

We present 3 cases of superior vena cava (SVC) syndrome following percutaneous right ventricular assist device (RVAD) placement. Each case underscores the importance of early recognition of SVC syndrome in patients with percutaneous RVAD insertion via the internal jugular vein and calls for heightened awareness of device-associated complications. (**Level of Difficulty: Advanced.**)

## Central Illustration

**Figure:**
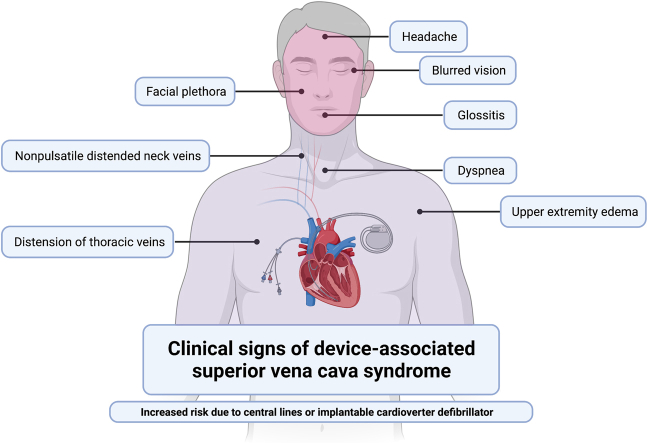

**Figure 1:**
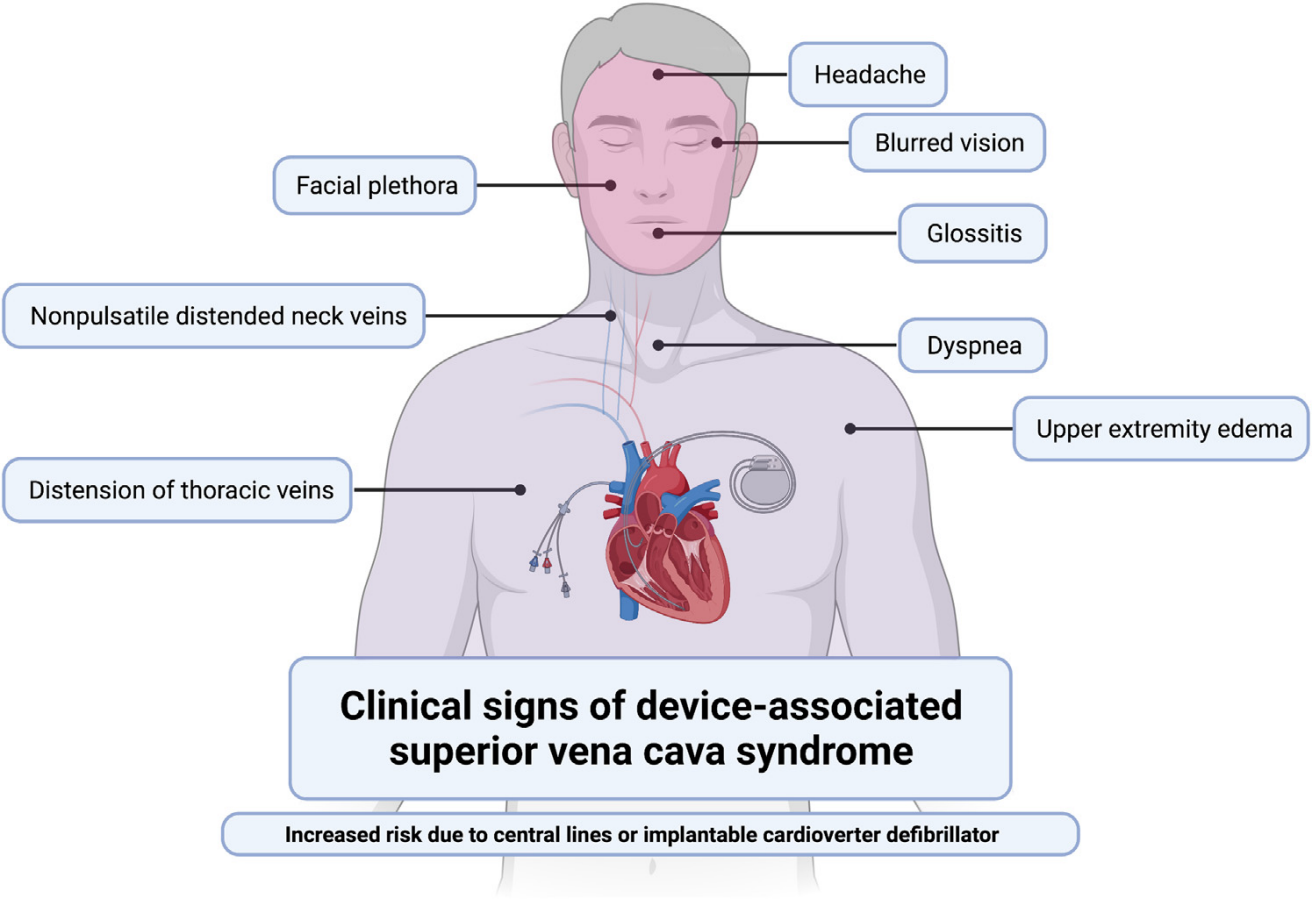
Clinical Signs of Superior Vena Cava Syndrome Device-associated venous congestion causes neurological, laryngopharyngeal, and upper extremity sequelae. Created with BioRender.com.

Superior vena cava (SVC) syndrome is caused by partial or complete occlusion of the SVC, and in recent years, the incidence of device-related SVC syndrome has risen because of modern-day use of catheters, pacemakers, and defibrillators. At present, device-related SVC syndrome accounts for up to 40% of all cases (1). Patients commonly present with facial and chest edema, dyspnea and cough, and nonpulsatile distended neck veins (Figure 1). Depending on the severity and location of the obstruction and the presence of collateral venous drainage, patients may also experience varying degrees of neurological (eg, headache, blurred vision), laryngopharyngeal (eg, glossitis), and upper extremity (eg, edema) sequelae (1). In this case series, we report 3 cases of SVC syndrome following implantation of the percutaneous dual-lumen ProTek Duo (Tandem Life) right ventricular assist device (RVAD). We review recent literature on postsurgical and iatrogenic causes of SVC syndrome and approaches for preventing and managing device-associated SVC syndrome.

**Learning Objectives**

• To recognize the development of SVC syndrome in patients with a dual-lumen percutaneous RVAD.
• To discuss predisposing risk factors to the development of a superior vena caval obstruction.
• To discuss management strategies in patients with SVC syndrome following placement of a dual-lumen percutaneous RVAD.

## Postsurgical and Iatrogenic Causes of SVC Syndrome

SVC syndrome can arise in postsurgical settings or as a consequence of iatrogenic obstruction to venous drainage. Currently, heart transplantation and ventricular assist devices may be used in end-stage heart failure that is unresponsive to interventional treatments. Although bicaval anastomosis during heart transplantation is preferred because of anatomic and hemodynamic benefits, this method effectively limits the distensibility of the SVC to the circumference of the suture line and may precipitate SVC syndrome with post-transplant RVAD use (2). Although uncommon, iatrogenic injury to the brachiocephalic venous system after heart transplant surgery can also lead to obstructed venous return.

Although both postsurgical and iatrogenic causes of SVC syndrome can develop secondary to a disruption in hemodynamic stability (eg, vena caval stenosis, hypercoagulability), scant literature has reported the onset of SVC syndrome after percutaneous RVAD cannulation via the right internal jugular vein (3). As the frequency of percutaneous RVAD support increases due to ease of use, it is critical to discuss recognition and management of device-associated SVC syndrome (4).

## Clinical Summary

### Patient 1

**Figure 2:**
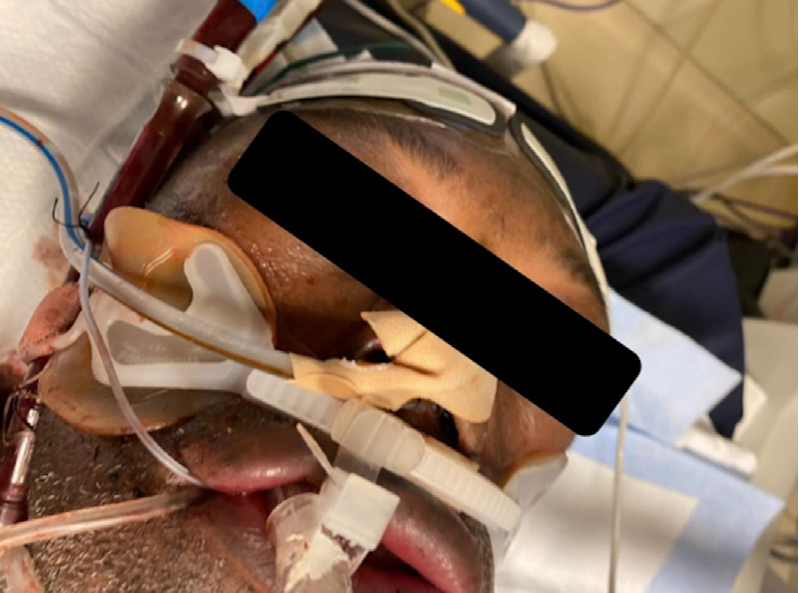
Superior Vena Cava Syndrome Presentation After Percutaneous Cannulation Patient 1 presented with massive facial edema and significant swelling of the upper extremities and central venous pressure of 45 mm Hg.

A 61-year-old man presented with restrictive cardiomyopathy with biventricular failure and underwent orthotopic heart transplantation (OHT). The patient had a dual-chamber implantable cardioverter-defibrillator (ICD) that was removed following OHT. A postoperative transesophageal echocardiogram (TEE) revealed a moderately dilated right ventricle (RV) with normal function. Postoperative day 2, the patient became profoundly hypotensive and unresponsive to vasopressors, and because of high central venous pressure (CVP) and RV dysfunction, the patient underwent RVAD extracorporeal membrane oxygenation. Approximately 30 hours later, the patient had marked swelling of the head and upper extremities and a CVP of 45 mm Hg (Figure 2). The patient underwent immediate surgery for explantation of the percutaneous RVAD and was converted to a central surgical RVAD system, which was indicated following refractory RV failure and SVC syndrome. After management, signs of SVC syndrome resolved, and the patient survived to discharge.

### Patient 2

A 55-year-old man presented with acute decompensated left ventricular systolic heart failure and cardiogenic shock that required an intra-aortic balloon pump; he underwent LVAD and RVAD implantation. One week before LVAD placement, the patient’s biventricular ICD was removed because of sepsis. Shortly after, central RVAD support was switched to percutaneous support with no complications. Approximately 45 hours following placement of the device, the patient exhibited rapidly progressive head and neck swelling suggestive of SVC syndrome. After removal of a preexisting left internal jugular central venous line, the patient experienced improved facial edema, and signs of SVC syndrome resolved.

### Patient 3

**Figure 3:**
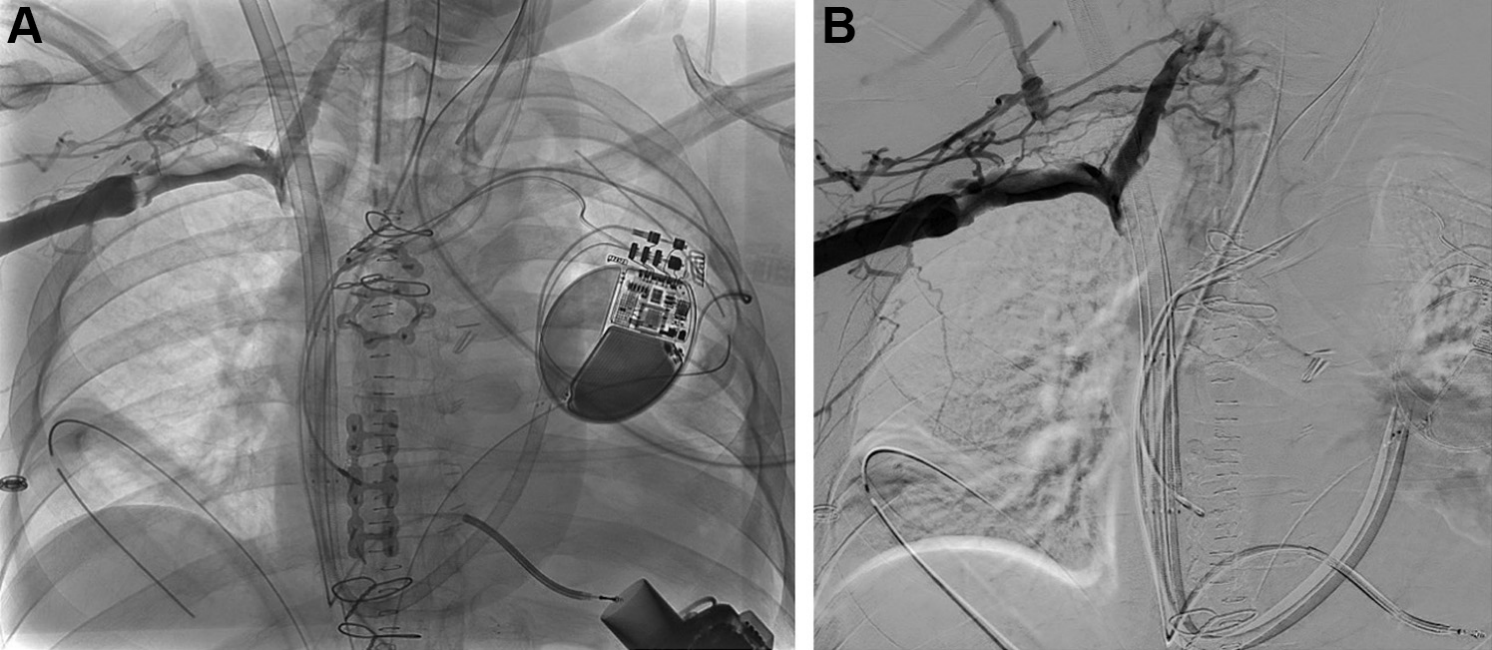
Venography at the Time of Clinical Signs of Superior Vena Cava Syndrome **(A)** Venogram revealed near-occlusion of the superior vena cava after device implantation. **(B)** Digital subtraction venography shows prominent collateralization proximal to the occlusion.

A 50-year-old man with a history of HeartMate II explant for recovery presented with recurrence of heart failure with a reduced left ventricular ejection fraction of 10% and associated RV dysfunction. After implantation of LVAD and RVAD support, the patient experienced facial edema, orbital swelling, and underwent emergent venogram that showed near occlusion of the SVC (Figure 3, Videos 1 and 2). Despite clinical improvement during the subsequent 3 hours, RVAD support was removed, and signs of SVC syndrome resolved shortly thereafter. The patient had a dual-chamber ICD during RVAD placement and removal, which might have contributed to reduced SVC distensibility.

## Diagnosis and Management

The diagnosis of SVC syndrome is accomplished using clinical picture and supplementary imaging modalities like chest radiography, contrast-enhanced computed tomography scanning, duplex ultrasound, conventional catheter-based digital subtraction venography, and magnetic resonance venography (1). These tools grade the severity of the syndrome, which is critical for informing initial management of the patient.

For nonmalignant causes of SVC syndrome, including placement of a dual-lumen cannula, there are several strategies for preventing venous congestion. In the setting of transplant, preoperative SVC imaging can reveal anatomical variance in caval diameters; significant discrepancy between host and donor may increase risk for bicaval stenosis and subsequent venous congestion. Likewise, we suggest performing a venogram before RVAD placement in patients with preexisting leads or central lines because it may reveal subclinical stenosis. Similarly, venous obstruction can be circumvented via imaging of the SVC-RA junction among patients before device placement; however, this approach poses a significant challenge because of poor validation of ranges for cross-sectional radiographic sizing across different imaging modalities (5).

In addition, the SVC is a compliant vasculature, and its sizing is likely dependent on the hemodynamic status at the time of measurement. More informative imaging techniques may be accomplished via computed tomography venography, but this approach carries a substantial radiation dose and is poorly validated because venous size chiefly depends on intrathoracic pressure and volume status (6). An alternative monitoring technique is to measure CVP through percutaneous catheterization via the left internal jugular or brachiocephalic vein using a 4-F to 7-F catheter. If high CVP is observed despite proper device placement, clinical vigilance might be key to identifying development of SVC syndrome. At our institution, SVC syndrome was noted in 3 of 22 (13.6%) device implants. We hypothesize that design modifications to facilitate drainage from the portion of the RVAD cannula that is proximal to the cavo-atrial junction can provide adequate decompression.

Venous scarring from preexisting intravascular leads may result in reduced distensibility and may precipitate venous congestion after percutaneous RVAD cannulation. For example, SVC syndrome following pacemaker implantation can occur secondary to the formation of vegetations or via thrombosis after endothelial disruption (7). In all 3 cases, the patients had longstanding intravascular leads in place, and 2 patients had leads removed before cannulation. Moreover, we noted development of SVC syndrome within 48 hours in each patient. Ultimately, if there is concern for SVC syndrome following the use of percutaneous RVAD support, central surgical RVAD conversion can be executed and managed per best practices.

## Conclusions

Here, we describe 3 cases of SVC syndrome following percutaneous dual-lumen cannulation for extracorporeal life support. Patients with signs of SVC syndrome, including facial and chest edema, dyspnea and cough, and nonpulsatile distended neck veins, experienced improvement following either device explantation or removal of the accompanying central venous catheter within the left internal jugular and subclavian venous system. By considering multiple etiologies for SVC syndrome, we hypothesize that appropriate RV support and early recognition of SVC syndrome are critical for preventing venous obstruction.

## Funding Support and Author Disclosures

The authors have reported that they have no relationships relevant to the contents of this paper to disclose.

## Appendix

Supplemental Video 1 Right branch venography demonstrating SVC syndrome within 24 hours. Video of fluoroscopic venogram from right branches revealed near-occlusion of the SVC.

Supplemental Video 2 Left branch venography demonstrating SVC syndrome within 24 hours. Video of fluoroscopic venogram from left branches revealed near-occlusion of the SVC.
